# The longitudinal relationship between job mobility, perceived organizational justice, and health

**DOI:** 10.1186/1471-2458-8-164

**Published:** 2008-05-19

**Authors:** Mats Liljegren, Kerstin Ekberg

**Affiliations:** 1 National Centre for Work and Rehabilitation, Department of Medicine and Health Sciences ,Linköping University, SE-581 83 Linköping, Sweden

## Abstract

**Background:**

The main purpose of the present study was to examine the 2-year longitudinal and reciprocal relationship between job mobility and health and burnout. A second aim was to elucidate the effects of perceived organizational justice and turnover intentions on the relationship between job mobility (non-, internally and externally mobile), and health (SF-36) and burnout (CBI).

**Methods:**

The study used questionnaire data from 662 Swedish civil servants and the data were analysed with Structural Equation Modeling statistical methods.

**Results:**

The results showed that job mobility was a better predictor of health and burnout, than health and burnout were as predictors of job mobility. The predictive effects were most obvious for psychosocial health and burnout, but negligible as far as physical health was concerned. Organizational justice was found to have a direct impact on health, but not on job mobility; whereas turnover intentions had a direct effect on job mobility.

**Conclusion:**

The predictive relationship between job mobility and health has practical implications for health promotive actions in different organizations.

## Background

The mobility of employees has a considerable effect on an organization's productivity and competitiveness. Job mobility also has a significant impact on the individual employee's situation, with regard to wages and career [1] work characteristics such as autonomy and task diversity [2] organizational commitment [3] and motivation for professional development [4]. Job mobility is furthermore affected by a number of individual and organizational factors such as gender and marital status [5] organizational commitment and job satisfaction [2,6] and social support and organizational justice [6].

Few earlier studies have elucidated the relationship between job mobility and health and the causal relationship between job mobility and health is somewhat of a scientific terra incognita. Two possible causal directions are possible: job mobility could constitute a predictor to health and burnout or health and burnout could act as predictors to job mobility.

### Job mobility as predictor to health and burnout

The relationship between employee mobility in terms of organizational mobility (change of position in an organizational system i.e. promotion or degradation) and health has been examined in detail during recent decades [7]. Bartley and Plewis [8] found that employed men who had been downwardly mobile (mobility between different occupational social classes) were more likely to report long-term illness than men who were upwardly mobile. Ribet, Zins, Gueguen, Bingham, Goldberg, Ducimetière and Lang [9] studied the effects of promotion on health related risk factors. The results showed that non-mobile had a higher risk of becoming smokers, excessive alcohol consumers and hypertensive than upwardly mobile men.

While the relationship between downward or upward mobility in organizations and health has been investigated in various studies, the relationship between job mobility (in terms of individual change of workplace or organization) and health has not been studied to any considerable extent, and the results provide an unclear picture. Metcalf, Davey-Smith, Sterne, Heslop, Macleod and Hart [10] found a positive association between frequent job changes and risk behaviours for health, such as smoking, alcohol consumption and sedentary lifestyle. No relationships were however found between frequent job changes and health (measured as BMI, diastolic blood pressure, forced expiratory volume and plasma cholesterol level). Liljegren and Ekberg [11] found longitudinal differences between non-mobile and respondent groups who had left the organization regarding their degree of personal and work-related burnout: the externally mobile group showed statistically significant reduction of the degree of burnout compared with the non-mobile group.

### Health and burnout as predictor to job mobility

Koeske and Kirk [12] found that psychological well-being did not predict actual turnover within 18 months but Ribet, Zins, Gueguen, Bingham, Goldberg, Ducimetière and Lang [9] found a longitudinal association between health related risk factors and job mobility since smokers and excessive alcohol drinkers had a higher risk of non-mobility than non-smokers and non excessive alcohol drinkers. Fields, Dingman, Roman and Blum [13] found that stress was associated with increased likelihood of moving to the same job in a different organization (external mobility), but they found no association between job stress and likelihood of moving to a different job in the same organization (internal mobility). In an extensive metaanalysis, Mor Barak, Nissly and Levin [6] found statistically significant positive associations between burnout and external job mobility.

### Turnover intentions

Turnover intentions, i.e. an intention or desire to change job, is closely associated with job mobility. The predictive effect of turnover intentions on actual behaviour has been studied extensively and proven in a number of studies [14-17]. Aronsson and Göransson [18] found that employees who wanted to change jobs but did not put their intentions into practice reported more symptoms such as headache, slight depression and fatigue than other employees i.e. an interaction effect between turnover intentions and job mobility due to their effect on health. Liljegren and Ekberg [11] found no support for this interaction effect in their results.

### Organizational justice

Turnover intentions are associated with low perceptions of perceived organizational distributive, procedural and interactional justice [19,20]. Schmitt and Dörfel [21] showed that perceived procedural injustice at work was negatively correlated with job satisfaction and psychosomatic well-being. Elovainio, Kivimäki and Vahtera [22] found a positive correlation between high organizational justice and high self-rated health, fewer minor psychiatric disorders, and less sickness absence. Liljegren and Ekberg [23] found a longitudinal positive association between organizational justice, and better self-rated health and fewer burnout symptoms. Based on these studies, it may be assumed that organizational justice is one of the mediating factors in the relationship between job mobility and health.

To summarize, the literature shows that job mobility is associated with health and burnout. Even if the earlier empirical results are contradictory they, in most cases, emanate from the assumption that mobility predicts health. The reversed causal direction, that health could affect job mobility, is largely overlooked. This is rather surprising, since one of the most controversial discussions within the field of social mobility and health concerns whether health should be considered as a consequence or a cause of social mobility. From the health selection or drift hypothesis [7] it may be assumed that health has a causal effect on the individual's chances regarding job mobility. According to this hypothesis, a person with worse health is less able to take action towards job mobility than a person who is in good health. A number of factors are unclear, however. Is health an incentive for the individual to move into a new position, or is mobility a health-promoting factor? What promotes the individual's turnover intentions? During recent years a number of studies have shown that burnout, as a measure of bad health, is associated with organizational and psychosocial factors at work, and that perceived justice is one important determinant. This indicates that turnover intentions may be based on negative, rather than positive, motivation.

The main purpose of the present study is to examine the longitudinal relationship between job mobility and health, in particular the reciprocal relation between health and job mobility. Possible distinctions between the effects of job mobility on different aspects of health (i.e. physical health, psychosocial health and burnout) and the effect of perceived organizational justice, age and turnover intentions on the relationship between job mobility and health will be elucidated.

## Methods

### Sample and procedure

A questionnaire was sent by post to all (n = 1010) employees (including those on the sick list and on leave of absence) at three different regional organizations of the Swedish National Labour Market Administration (AMV). A majority were working as employment officers in different local employment agencies. The mean age was 48.7 years, ranging from 25 to 65 years. Of the 1010 employees, 602 (59.6%) were women and 408 (40.4%) were men. In all, 792 subjects (78.4% of the total population) responded to the questionnaire.

Two years after the first questionnaire was sent a follow-up questionnaire was distributed to those employees who had responded to the first questionnaire, including those who had left the organization during the period (due to turnover or retirement). The respondents who had retired between the baseline and follow-up (n = 15) were excluded from the subsequent analysis. The follow up questionnaire was answered by 662 subjects, 65.5% (disregarding the retiring subjects who had retired).

The final study population consisted of the subjects who had responded to the first and second-wave questionnaire and who had not retired during the study period (n = 662). The mean age of the final study population was 49.4, ranging from 27 to 64 years: 401 (60.6%) were women and 261 (39.4%) were men.

The Ethics Committee at Linköping University approved the study.

### Measures

#### Demographical variables

Sex and age (at baseline) were used as demographical variables in the subsequent analyses, as earlier studies report that health, in particular physical health, is strongly associated with health, and that women tend to report poorer health than men (see for example, Sullivan and Karlsson, [24]).

#### Perceived organizational justice

The individual experience of justice was measured by three different self-assessment instruments. Distributive justice was measured by a five-item instrument [25] The response scale was a five-point Likert scale (1 = very fair, 5 very unfair) (example item: *"How fair has the organization^1 ^been in rewarding you when you consider the responsibilities you have?")*. Procedural justice was measured by a four-item instrument [26]. The response scale was a five-point Likert scale (1 = strongly disagree, 5 = strongly agree) (example item: *"The organization went about deciding to reorganize^2 ^in a way that was not fair to me")*. Interactional justice was measured by six items and five-point Likert response scales (1 = strongly disagree, 5 = strongly agree) [27] (example item: *"Your supervisor considered your viewpoint")*. The Swedish versions of the three instruments have been used in earlier studies and have showed internal-consistency reliability coefficients (Cronbach's alpha) above .85 [28].

#### Turnover intentions

Turnover intentions at baseline were measured using the exit subscale from a modified EVLN-typology instrument [19]. In addition to the original validation of the instrument, performed by Hagedoorn, van Yperen, van der Vliert and Buunk [19], the psychometric properties of the Swedish version of the instrument have been tested [28]. The internal consistency was high (Cronbach's alpha: .90) and there was a strong association between exit behavioural response and actual exit behavior, indicating a high degree of predictive validity. The subscale used consists of 6 items (Initial statement: *"Would you indicate how likely it is that you would react to problematic events [at work] in the described ways" *example item: *"Consider possibilities to change job")*. The response scale was a seven-point Likert scale (ranging from "definitely not" to "definitely yes").

#### Job mobility

Information about actual turnover behaviour was provided from the organizations where the respondents were employed. Job mobility was coded as 1: non-mobile (still at original employment), 2: internal mobile (changing workplace but still within the organization) and 3: external mobile (changing workplace and organization).

#### Health

Overall self-rated health was measured using the SF-36 [29]. The SF-36 is a 36-item instrument measuring eight different health concepts: physical functioning (PF), role limitations due to physical problems (RP), bodily pain (BP), general health perceptions (GH), vitality (VT), social functioning (SF), role limitations due to emotional problems (RE), and general mental health (MH). The first four dimensions are considered as primarily measuring physical aspects of health and the remaining four scales measure mental or psychosocial aspects of health [30]. All scales range from 0 (worst) to 100 (best). A detailed description of items, score derivation, translation and validation for the SF-36 scales is found in Sullivan, Karlsson and Ware [30].

#### Burnout

The degree of burnout was measured by the Copenhagen Burnout Inventory, CBI, [31]. The inventory consists of three scales measuring different dimensions of burnout: personal burnout (six items), work-related burnout (seven items) and client-related burnout (six items). All items have five response alternatives ranging from 'always/very high degree' (coded as '100') to 'never/very low degree' (coded as '0') with the intervening alternatives coded as '75', '50' and '25', (example item: *"How often do you feel tired?")*. A summary score for each response dimension was calculated as the average value of the individual item scores. A high score indicates a high degree of burnout.

### Statistical analyses

As a first step in the analysis, the distribution of turnover intentions, perceived organizational justice, job mobility and self-rated health and burnout at baseline were analyzed in relation to the demographical variables sex and age, using t-test and ANOVA (corrected for multiple comparisons with Bonferroni correction). As earlier studies have shown that both organizational justice and turnover intentions have a distinct and clear relationship with health, burnout and job mobility, turnover intentions and perceived organizational justice were used as independent or exogenous variables in the tested model.

Secondly, correlations (Spearman's rank correlation coefficients) between sex, age, turnover intentions, job mobility, self-rated health and burnout were computed.

As a third step in the analysis, a structural equation model, SEM, was formulated and tested. The model used age, distributive, procedural and interactional organizational justice (at baseline), turnover intentions (at baseline) self-rated health (at baseline), burnout (at baseline) and job mobility as exogenous variables. The endogenous variables in the analysis consisted of self-rated health (at baseline and follow-up), burnout (at baseline and follow-up) and job mobility.

Self-rated health, at baseline and follow-up, was measured by two different latent variables: physical health indicated by the SF-36 variables PF, RP, BP and GH: and psychosocial health, indicated by the SF-36 variables VT, SF, RE and MH. Sex was deleted in this analysis due to its two-category response format since binary data can be difficult to analyze with SEM [32], requiring either very large sample sizes for asymptotic least squares or integration of the multivariate normal distribution over as many dimensions as there are relatives in the pedigree [33]. Longitudinal relationships between the same variable and correlations between residual variables for health, justice and burnout were inserted in the model.

Incomplete data was handled by using the maximum likelihood estimation approach, i.e. treatment of missing data assumption of multivariate normality, based on the direct maximation of the likelihood of the observed data. This approach has numerous advantages over other methods to treat missing data as listwise or pairwise deletion. Firstly, the ML estimation is theory based and not, as many other methods, ad-hoc solutions. Secondly, where the unobserved values are missing completely at random the deletion approach is consistent but not efficient (in the statistical sense): the ML approach is both consistent and efficient. Where the observed values are only missing at random, deletion estimates could be biased, ML estimates are asymptotically unbiased [32]. Root Mean Square Error of Approximation, RMSEA, was calculated for the tested model. Based on recommendations from Bentler [34] and Marsch, Balla, & Hau [35], the RMSEA were complemented with three relative goodness-of-fit indices: the Non-Normed Fit Index (NNFI), the Incremental Fit Index (IFI), and the Comparative Fit Index (CFI). Values of .90 or higher are considered to indicate a good fit for the relative indices [36]. Byrne [32] proposed an RMSEA of ≤ .05 for good model fit, Hu and Bentler [37] advocate a ≤ .06 limit, and Browne and Cudeck [38] a ≤ .08 limit for acceptable fit.

SPSS version 14.0 and AMOS version 6.0 were used for the statistical analyses.

## Results

The descriptive statistics (means and standard deviations) of turnover intentions, perceived organizational justice, job mobility (number and percent), self-rated health and burnout at baseline according to sex and age, are presented in table 1.

**Table 1:** Descriptive statistics for the included variables

		**Sex**		**Age**				**Total**
		Women	Men	-34	35–44	45–54	55-	
**Turnover intentions**		3.55 *(1.12)*	3.70 *(1.15)*	4.22*(1.03)*	4.07 *(1.00)*	3.76 *(1.03)*	3.05 *(1.10)*	3.61 *(1.14)*
								
**Perceived organizational justice**	Distributive justice	3.01 *(0.96)*	3.03 *(0.91)*	2.72 *(0.93)*	2.93 *(0.95)*	2.96 *(0.92)*	3.19 *(0.91)*	3.02 *(0.94)*
	Procedural justice	3.38 *(0.88)*	3.43 *(0.94)*	3.36 *(0.73)*	3.42 *(0.82)*	3.39 *(0.97)*	3.40 *(0.92)*	3.40 *(0.90)*
	Interactional justice	3.95 *(0.71)*	3.90 *(0.79)*	3.82 *(0.78)*	3.89 *(0.75)*	3.95 *(0.76)*	3.95 *(0.72)*	3.93 *(0.74)*
								
**Job mobility**	Non-mobile	306 *(76.3%)*	179 *(68.6%)*	32 *(58.2%)*	99 *(73.3%)*	174 *(74.1%)*	180 *(76.0%)*	485 *(73.3%)*
	Internal mobile	46 *(11.5%)*	42 *(16.1%)*	8 *(14.5%)*	10 *(7.4%)*	36 *(15.3%)*	34 *(14.3%)*	88 *(13.3%)*
	External mobile	49 *(12.2%)*	40 *(15.3%)*	15 *(27.3%)*	26 *(19.3%)*	25 *(10.6%)*	23 *(9.7%)*	89 *(13.4%)*
								
**SF-36**	Physical functioning	88.0 *(16.9)*	92.2 *(12.4)*	92.7*(16.1)*	93.2 *(12.1)*	90.1 *(16.1)*	86.5 *(15.6)*	89.7 *(15.4)*
	Role limitations (physical)	78.0 *(35.1)*	82.7 *(30.6)*	80.0 *(35.8)*	83.9 *(28.4)*	79.5 *(33.7)*	78.1 *(35.3)*	79.9 *(33.5)*
	Bodily pain	70.6 *(27.7*)	75.7 *(24.0)*	77.8 *(23.6)*	76.7 *(23.5)*	71.4 *(26.9)*	70.3 *(27.7)*	72.6 *(26.4)*
	General health	70.0 *(23.2)*	72.5 *(19.7)*	75.7 *(19.4)*	72.0 *(20.8)*	73.0 *(20.8)*	67.5 *(23.7)*	71.0 *(21.9)*
	Vitality	56.0 *(23.1)*	64.2 *(20.9)*	56.5 *(20.8)*	57.5 *(21.1)*	58.9 *(23.4)*	61.3 *(23.0)*	59.3 *(22.6)*
	Social functioning	74.7 *(26.6)*	81.7 *(21.7)*	80.2 *(19.0)*	78.1 *(23.1)*	76.1 *(26.8)*	77.9 *(25.4)*	77.5 *(25.0)*
	Role limitation (emotional)	77.8 *(35.0)*	83.4 *(30.0)*	84.2 *(30.7)*	77.2*(34.7)*	79.1 *(32.4)*	81.5 *(33.7)*	80.0 *(33.2)*
	Mental health	73.4 *(18.7)*	77.2 *(17.1)*	75.3 *(15.0)*	74.2 *(18.0)*	74.1 *(18.5)*	76.1 *(18.7)*	74.9 *(18.2)*
								
**CBI**	Personal burnout	46.6 *(19.2)*	40.4 *(18.3)*	43.4 *(19.0)*	46.9 *(18.4)*	44.6 *(19.1)*	42.1 *(19.4)*	44.1 *(19.1)*
	Work-related burnout	40.2 *(20.2)*	35.8 *(18.2)*	38.3 *(18.0)*	40.0 *(19.1)*	39.3 *(19.9)*	36.6 *(19.8)*	38.4 *(19.6)*
	Client-related burnout	36.3 *(20.3)*	36.2 *(18.0)*	36.9 *(18.9)*	36.8*(21.2)*	37.9 *(19.5)*	34.0 *(18.1)*	36.2 *(19.4)*
	***Total***	***401***	***261***	***55***	***135***	***235***	***237***	***662***

Descriptive statistics for turnover intentions at baseline, perceived organizational justice (means and standard deviations), occupational mobility (numbers and percent), self-rated health (SF-36) and burnout (CBI) (means and standard deviations), distributed among age and sex groups.

Turnover intentions (*F*(3,642)*= *37.86, *p *< .001) and distributive justice (*F*(3,643)*= *5.34*, p *= .001) differed between age categories: the youngest respondents had a higher degree of turnover intentions and lower perceived distributive justice than the older respondents. Women had significantly lower health in the SF-36 variables PF (*t*(646) = 3.43, *p *< .001) BP (*t*(644) = 2.43, *p *< .001), SF (*t*(645) = 3.48, *p *< .001) and RE (*t*(644) = 2.12, *p *< .001). Physical function, SF-36 variable PF, decreased with increasing age (*F*(3,644)*= *6.54, *p *< .001).

During the study period 485 (73%) subjects remained at the same workplace, i.e. they were "non-mobile": 88 (13%) subjects changed workplace but remained within the same organization, i.e. they were "internally mobile"; and 89 (14%) subjects left the organization, i.e. they were "externally mobile". The younger respondents tended to be more inclined to external mobility than the older respondents. A less evident difference was found between the sexes: men tended to be slightly more inclined to job mobility than women.

The correlations between the included variables were computed. The results are presented in table 2.

**Table 2:** Correlations between the included variables

		Sex	Age	Organizational justice			Turnover intentions	Job mobility	Health (SF-36) Follow-up								Burnout (CBI) Follow-up		
				Distributive	Procedural	Interactional			PF	RP	BP	GH	VT	SF	RE	MH	Personal	Workrel.	Clientrel.
Sex		-	-	-	-	-	-	-	-.14**	-.12**	-.10*	-.05	-.14**	-.09*	-.07	-.07	.16***	.10*	-.05
Age		-.04	-	-	-	-	-	-	-.23***	-.01	-.05	-.04	.15***	.10*	.10*	.13**	-.10*	-.11*	-.15***
Justice	Distrib.	-.02	.13**	-	-	-	-	-	.04	.10*	.11**	.14***	.24***	.22***	.22***	.18***	-.27***	-.32***	-.25***
	Proced.	-.03	.00	.54***	-	-	-	-	.11**	.14**	.18***	.22***	.23***	.21***	.20***	.17***	-.26***	-.30***	-.21***
	Interact.	.02	.04	.53***	.50***	-	-	-	.09*	.17***	.16***	.19***	.19***	.19***	.19***	.17***	-.21***	-.28***	-.19***
Turnover int.		-.06	-.42***	-.26***	-.23***	-.16***	-	-	.15***	-.03	.01	-.05	-.15**	-.20***	-.16***	-.23***	.16***	.22***	.23***
Job mob.		-.08*	-.09*	.04	.06	-.02	.13**	-	.05	.03	.07	.07	.08	.05	.01	.09*	-.12**	-.12**	-.04
Health (SF-36) Baseline	PF	-.12**	-.26***	.03	.11**	.09*	.10*	.04	.59***	.32***	.42***	.48***	.31***	.27***	.11**	.19***	-.24***	-.15**	.00
	RP	-.05	-.04	.12**	.15***	.10*	-.06	.03	.32***	.32***	.31***	.30***	.28***	.28***	.19***	.18***	-.26***	-.20***	-.05
	BP	-.08*	-.08	.07	.19***	.15***	-.01	.03	.42***	.34***	.51***	.40***	.31***	.27***	.18***	.23***	-.33***	-.24***	-.09*
	GH	-.03	-.09*	.15***	.27***	.24***	-.09*	.07	.46***	.37***	.48***	.66***	.44***	.36***	.19***	.35***	-.42***	-.35***	-.16***
	VT	-.17***	.10*	.29***	.30***	.26***	-.15***	.06	.31***	.34***	.37***	.49***	.59***	.45***	.32***	.46***	-.55***	-.49***	-.28***
	SF	-.11**	.03	.28***	.30***	.24***	-.18***	.00	.26***	.33***	.34***	.40***	.43***	.48***	.35***	.38***	-.45***	-.44***	-.22***
	RE	-.07	.04	.20***	.21***	.17***	-.13**	.01	.18**	.23***	.22***	.24***	.30***	.30***	.29***	.28***	-.32***	-.32***	-.14**
	MH	-.10*	.08*	.25***	.26***	.26***	-.15***	.03	.20***	.24***	.32***	.39***	.47***	.43***	.32***	.55***	-.47***	-.44***	-.26***
Burnout (CBI) Baseline	Personal	.16***	-.09*	-.31***	-.35***	-.31***	.21***	.00	-.26***	-.36***	-.38***	-.45***	-.55***	-.44***	-.35***	-.46***	.67***	.62***	.38***
	Workrel.	.11*	-.09*	-.39***	-.39***	-.36***	.26***	.01	-.18***	-.28***	-.28***	-.38***	-.50***	-.42***	-.35***	-.45***	.60***	.64***	.45***
	Clientrel.	-.01	-.08*	-.25***	-.29***	-.21***	.24***	-.03	-.10*	-.16**	-.19***	-.24***	-.29***	-.24***	-.20***	-.30***	.39***	.43***	.61***

Correlations (Spearman's correlation coefficients) between sex, age, perceived organizational justice, turnover intentions, health (SF-36) and burnout (CBI) at baseline and follow-up. * .01 ≤ p < .05 ** .001 ≤ p < .01 *** p < .001

Negative, longitudinal associations were found between age and one of the SF-36 variables: 'physical functioning', (PF), and all three burnout variables. Positive associations were found between age and the psychosocial SF-36 variables 'vitality' (VT), 'social functioning' (SF), 'role limitations due to emotional problems' (RE) and 'mental health' (MH).

Perceived organizational justice at baseline was positively associated with both physical and psychosocial health, except for the association between distributive justice and physical functioning, and negatively associated with burnout, both at baseline and at the two-year follow-up. Turnover intentions at baseline were associated with low perceived organizational justice, low psychosocial health and a high degree of burnout.

Health (both psychosocial and physical) at baseline showed positive associations with health and negative associations with burnout at the follow-up. Burnout at baseline showed negative associations with health and positive associations with burnout at follow-up.

To analyse the causal relationships between job mobility, and health and burnout, structural equation modelling (SEM) statistical technique was used. The indices for the model were χ^2^/df (2369.381/261) = 9.078, RMSEA = .111, CFI = .823, IFI = .826, and NNFI = .725.

**Figure 1:**
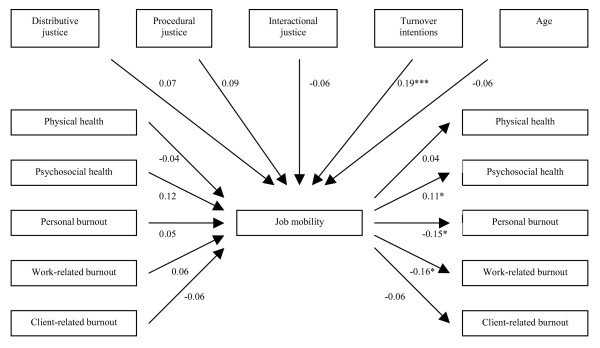
**Schematic representation of the main results of the SEM analysis (Standardized maximum likelihood estimates and p-values).** * .01 ≤ p < .05 *** p < .001.

The only exogenous variable that significantly predicted job mobility was turnover intentions. Job mobility, as an exogenous variable, predicted psychosocial health, personal and work-related burnout at the follow-up (Figure 1). Turnover intentions had a direct causal effect on job mobility: a high degree of intention to quit affects actual turnover, while according to the results neither age nor perceived organizational justice have any direct effect on job mobility.

All the results from the SEM analysis are presented in table 3.

**Table 3:** Results of the SEM

	Physical health (baseline)	Psychsocial health (baseline)	Personal burnout (baseline)	Work-related burnout (baseline)	Client-related burnout (baseline)	Physical health (follow-up)	Psychsocial health (follow-up)	Personal burnout (follow-up)	Work-related burnout (follow-up)	Client-related burnout (follow-up)	Job mobility
Age	-0.13**	0.01	-0.03	0.02	0.00	0.04	0.09*	-0.07*	-0.04	-0.06	-0.06
Distributive justice (baseline)	-0.03	0.16**	-0.13**	-0.19***	-0.10*	0.10*	0.06	-0.09*	-0.09*	-0.09*	0.07
Procedural justice (baseline)	0.22***	0.23***	-0.22***	-0.22***	-0.21***	-0.06	-0.06	0.04	0.05	0.02	0.09
Interactional justice (baseline)	0.12*	0.09	-0.13**	-0.14**	-0.02	0.05	-0.01	0.03	-0.03	0.03	-0.06
Turnover intentions (baseline)	0.02	-0.05	0.08*	0.15***	0.19***	0.01	-0.08*	0.01	0.06	0.04	0.19***
Physical health (baseline)	-	-	-	-	-	1.09***	0.20**	-0.19***	-0.07	0.11	-0.04
Psychosocial health (baseline)	-	-	-	-	-	-0.39***	0.25***	0.02	-0.01	0.02	0.12
Personal burnout (baseline)	-	-	-	-	-	-0.20**	-0.17*	0.58***	0.26***	0.04	0.05
Work-related burnout (baseline)	-	-	-	-	-	0.06	-0.16	0.01	0.32***	0.15*	0.06
Client-related burnout (baseline)	-	-	-	-	-	-0.04	0.03	0.05	0.05	0.50***	-0.06
Job mobility	-	-	-	-	-	0.04	0.11**	-0.15***	-0.16***	-0.06	-

Standardized maximum likelihood estimates and p-values between exogenous variables versus endogenous variables.* .01 ≤ p < .05 ** .001 ≤ p < .01 *** p < .001

To sum up, the results showed that job mobility was a more distinct predictor of health and burnout, than health and burnout were as predictors of job mobility. Job mobility predicted better psychosocial health and less personal and work-related burnout. Turnover intentions predicted job mobility. None of the three forms of organizational justice predicted job mobility.

## Discussion

The results of the present study are, partially, in line with those of earlier studies. The relationship between mobility and increased health adds to the results of Ribet, Zins, Gueguen, Bingham, Goldberg, Ducimetière and Lang [9] and Liljegren and Ekberg [11] and makes it possible to assume that the effect of job mobility, i.e. change of workplace, on individual health is similar to that of upward hierarchal mobility. One plausible explanation is that for the majority of individuals, job mobility could be experienced as professional development or even the result of an actual promotion.

The results reported by Fields, Dingman, Roman and Blum [13] and Mor Barak, Nissly and Levin [6], underlining the suggestion that health promotes mobility, found no support in the present study. Ribet, Zins, Gueguen, Bingham, Goldberg, Ducimetière and Lang [9] showed a dual-reciprocal association between organizational mobility and health, whereas this dual association gained no support in the present study. The weak predictive effect of health and degree of burnout on job mobility raises a number of questions. There is no support in the present study for the assumption that low psychosocial health and a high degree of work-related burnout, are incentives for changing work or workplace. One possible explanation is that psychosocial ill health and burnout are characterized by symptoms such as apathy, exhaustion and resignation [39] which may restrict the individual's capacity to change work. The employment and work situation of individuals suffering from a high degree of physical ill health could also have restrictive effects on their intentions and their actual work mobility behaviour. Physical problems could be experienced as hampering factors, both by the employee him/herself and by a possible future employer, and could be perceived as disadvantages on a competitive labour market. It is reasonable to assume that these restrictive factors could counterbalance the desire to change jobs.

Perceived organizational justice showed no influence on job mobility in the multivariate analysis. However, the correlations between organizational justice and turnover intentions provide some, albeit weak, support for the hypothesis of justice as a mediating factor between job mobility and health.

Some methodological aspects are worthy of comment. The strengths of the present study are its longitudinal design and its relatively large study sample. Structural equation modelling, SEM, is based on maximum likelihood estimation, which assumes that the variables are continuous. One of the variables used, job mobility, is based on three different categories and could therefore be considered as a categorical scale but with some ordinal features. The use of SEM to analyse categorical data has been consistently discussed by Byrne [32]. Based on earlier studies she argues that categorical data could be treated as continuous, without any appreciable risk; however, some caution is required, if the number of categories are less than three, if the skewness is greater than 1, and if the skewness is differential (skewed in opposite directions). In the present analyses, the variable 'job mobility' has three ordinally arranged categories but also shows a moderately high skewness: 1.46. The skewness was, on the other hand, non-differential. According to Byrne [32], this could result in a risk of inflated χ^2 ^levels. In the present study this risk should be considered as relatively small when the result of the SEM analysis is largely confirmed by the initial correlation analysis but should still be considered when the results in the present study are interpreted.

Another methodological aspect worth of notice is the risk of non-response biases, especially when the present study elucidates such delicate topics as organizational justice and health, however, the relatively high dual-point-of-time response rate, 65.5%, speaks against this apprehension. Any regional effects are very probably negligible, due to the three organizational cohort design of the present study.

One topic that should be further problematized and developed in future research is the construct of job mobility. In the present study this complex construct is handled in a very simple way on a strictly descriptive level. The incentives and results of the process to change work are omitted. The same behaviour (i.e. to stay or go) could have very different outcomes depending on individual prerequisites and motives. A decision to change work could be the result of a desire to leave a monotonous and tedious job, or an opportunity to get a new job that is, hopefully, interesting and stimulating. The motive for changing jobs could be the result of a voluntary decision but also the result of an organizational transition process, which the individual is unable to influence. Another related aspect that influences both the desire to change and the actual chance to changing jobs is whether or not work is currently available: in periods of downsizing or of high unemployment the individual's decision is obviously influenced by external and societal factors.

## Conclusion

The main aim of the present study was to elucidate the longitudinal and reciprocal relationship between job mobility, and health and burnout. The two possible causal directions, job mobility as a predictor of health and burnout, and health and burnout as a predictor of job mobility, were elucidated. The results showed that job mobility was a considerably more distinct predictor of health and burnout, than health and burnout were as predictors of job mobility. The predictive effects that were found were obvious for psychosocial health and burnout, but negligible as far as physical health was concerned.

The results of the present study have some practical implications. Job mobility could be a method to improve health and decrease burnout, but for individuals in the relevant target group for health-promotive interventions, i.e. those with a low level of health and a high degree of burnout, the results show that job mobility is not likely. A subject for future research is therefore to identify restrictive factors as well as incentives that support job mobility, among the group with a low level of health and a high degree of burnout. A practical implication is also to develop health-promotive programmes that are based on increased job mobility but also consider underlying factors such as incentives and restrictions.

## Competing interests

The authors declare that they have no competing interests.

## Authors' contributions

ML: Study design, data collection, data analysis and writing the manuscript. KE: Study design and participation in writing the manuscript. All authors read and approved the final manuscript.

## Note

^1^"Hospital" in the original instrument

^2^"Move" in the original instrument

## Pre-publication history

The pre-publication history for this paper can be accessed here:


